# The efficacy of video-assisted thoracoscopic surgery lung biopsies in patients with interstitial lung disease: a retrospective study of 66 patients

**DOI:** 10.1186/1749-8090-9-45

**Published:** 2014-03-10

**Authors:** Dominic Morris, Vipin Zamvar

**Affiliations:** 1 College of Medicine and Veterinary Medicine, University of Edinburgh, The Chancellor’s Building, 49 Little France Crescent, Edinburgh EH16 4SB, UK; 2 Department of Cardio-thoracic Surgery, New Royal Infirmary of Edinburgh, 51 Little France Crescent, Edinburgh EH16 4SA, UK

**Keywords:** Interstitial lung disease, Video-assisted thoracoscopic surgery, Definite pathological diagnosis, Change in treatment, Usual interstitial pneumonia, Hypersensitivity pneumonitis

## Abstract

**Background:**

Diagnosing a specific type of Interstitial Lung Disease (ILD) is a challenging process and often necessitates that a Video-assisted Thoracoscopic Surgery (VATS) Lung Biopsy be performed. By analysing the proportion of patients who have their treatment changed after undergoing a VATS lung biopsy, this study aimed to determine the utility of performing this procedure in patients with ILD.

**Methods:**

The clinical data from sixty-six patients with suspected ILD, who underwent VATS lung biopsies at the New Royal Infirmary of Edinburgh (NRIE) in the period of 16th May 2011 – 11th February 2013, were analysed retrospectively. The main outcome measures considered in this study were: CT scan differential diagnoses, VATS lung biopsy histological differential diagnoses, post-VATS lung biopsy consensus diagnoses, 30-day mortality, surgical complications (minor and major), resultant changes in treatment and responses to these changes in treatment.

**Results:**

Following VATS biopsy a definite pathological diagnosis was made in 74.2% of cases. A change in treatment was initiated in 47.2% of patients, including in 80% of patients diagnosed with Hypersensitivity Pneumonitis and 60% of patients diagnosed with sarcoidosis. A positive response to treatment was experienced in 58% of patients whom underwent a change in treatment. Only 54% of patients who received a consensus diagnosis of UIP after VATS lung biopsy, had been given a differential diagnosis of “probable UIP” at CT scan. 15% of patients who received a differential diagnosis of “probable UIP” at CT scan, had their diagnosis changed to Hypersensitivity Pneumonitis after lung biopsy. There was one mortality (1.5%) in this series of patients and no other major complications. Minor complications to surgery were experienced in 28.8% of patients.

**Conclusions:**

This study highlights the effectiveness of performing VATS lung biopsies in patients with suspected ILD. The procedure leads to a change in treatment in almost half of all patients, including in the vast majority of cases of Hypersensitivity Pneumonitis. It also prevents what would be the inappropriate over-treatment of UIP. It has been shown to be a relatively safe procedure and thus, should be performed in all patients with suspected ILD, indeterminate in type from prior CT imaging.

## Background

Interstitial Lung Disease (ILD) denotes a collection of heterogeneous lung diseases which all primarily affect the interstitium of the lung. ILD most commonly presents with dyspnoea or a dry cough. Systemic features, such as weight loss or fatigue, are also common at presentation. On clinical examination, the patient will often be tachypnoeic and bibasal end inspiratory fine crackles may be heard on auscultation. Clubbing of the digits and cyanosis may occasionally occur in advanced disease and patients generally exhibit a restrictive pattern on Pulmonary Function Testing.

There are reported to be more than 200 different subtypes of ILD, so achieving a correct specific diagnosis is often challenging in a patient with ILD. This is of vital importance though, because the subtypes of ILD have different management protocols.

Computerised Tomography (CT) scanning is more specific than chest radiography in diagnosing subtypes of ILD [1]. One study reported CT scanning to carry a diagnostic accuracy of 61-80% [2]. Indeed, a CT scan which is highly typical of a particular type of ILD, may provide a physician with sufficient confidence to make the diagnosis without further investigation. For example, the presence of centrilobular nodules is highly typical of chronic Hypersensitivity Pneumonitis (HP). Conversely, the presence of basal, subpleural, reticular opacities, which are associated with honeycombing and traction bronchiectasis, would be highly typical of Usual Interstitial Pneumonia (UIP) and would probably preclude the need for further investigation. Indeed CT scanning has a diagnostic accuracy of over 90% in making the diagnosis of UIP, if radiological features are highly suggestive of the disease [3-6]. However, the CT scans of many cases of ILD, do not display features highly typical of a particular subtype of ILD; this is indeed the case in around 50% of cases of UIP [5,6].

When a diagnosis has been not achieved by CT imaging, patients can be investigated by bronchoscopy, with Bronchoalveolar Lavages (BALs) and Transbronchial Lung Biopsies (TBLBs) being performed. However, on balance, BAL is of limited utility in the diagnostic process for ILD, other than sometimes allowing for the exclusion of malignancy [7], or infection [8]. However on occasion it can be useful in detecting rare forms of ILD [9]. The diagnosing potential of TBLB is also unspectacular for many cases of ILD. A specific diagnosis is achieved in the range 29 - 79% of reported cases referred for the TBLB [10-14]. There are two main reasons for this: firstly, the procedure only allows the clinician to obtain a very small specimen of tissue and secondly, biopsies can only be obtained from within the peribronchial sheath.

A specific diagnosis remains unavailable to one third of all patients with ILD even after the procedures highlighted above have been conducted [15-18]. So for this sizeable group of patients, the only option remaining is for a Surgical Lung Biopsy (SLB). However, such an operation is not without its risks to the patient; indeed SLB does carry a slight risk of mortality. In recent years Video-assisted Thoracoscopic Surgery (VATS) has replaced the older, more invasive method, of performing a minithoracotomy in these patients.

The objective of this study was to assess the benefit of performing VATS to obtain a histological diagnosis in patients with ILD, indeterminate in type on CT imaging. The benefit was measured with respect to the diagnostic ability of this procedure in obtaining a Definite Pathological Diagnosis (DPD), as well as assessing the proportion of patients who have their treatment changed as a result of VATS being performed. Close attention was also paid to the safety of this procedure.

## Method

Seventy nine patients with suspected ILD, who consecutively underwent a VATS lung biopsy at the New Royal Infirmary of Edinburgh in the period between 16th May 2011 - 11th February 2013 were retrospectively analysed. Exclusion criteria included: patients whose likely diagnosis was malignancy at CT scan (5 patients) and patients whose CT scan results hadn’t been uploaded onto either Trak-care system or the Picture Archiving and Communication System (PACS) used by NHS Lothian (8 patients).

All data was obtained by inspection of patients’ medical records. The Lothian Regional Ethics Committee deemed that the study was a service evaluation and therefore did not require formal ethical review. The data collected included: patient demographics (age, gender etc.); presenting symptoms; differential diagnoses from CT scan; pulmonary function test (PFT) readings; pre-operative treatment; number, location and size of biopsy specimens; operative complications and length of hospital stay.

Two of the minor postoperative complications experienced in the patient cohort were “delayed wound healing” and “prolonged neuropathic pain”. Delayed wound healing was defined as the failure of surgical wounds to heal within 30 days of the operation. Prolonged neuropathic pain was defined as the failure of neuropathic pain to resolve within 30 days of the operation.

The decision to refer a patient for VATS lung biopsy would be made at a Multi-Disciplinary Team (MDT) meeting. VATS was carried out under general anaesthesia and single lung ventilation. Patients were intubated with a double lumen endobronchial tube and placed in the lateral decubitus position. The standard 3-port VATS technique was used. The thoracoscope was inserted into the first port site; this was created in the seventh intercostal space, somewhere between the anterior axillary line and the mid-axillary line. The locations of the two further port sites could then be determined under video-guidance. Biopsies were taken using an Endopath ETS 45 mm endoscopic linear cutter (Ethicon Endo-surgery, Cincinnati, OH). Choice of biopsy site was guided by CT imaging. The decision to take single or multiple biopsies was made by the operating thoracic surgeon. A single 32 F chest drain was inserted into one of the anterior port sites, with the other two port sites being closed with sutures. Biopsies were injected with formalin and immersed in a jar containing formalin, then sent to Pathology.

Further data was collected regarding: histological differential diagnoses; consensus diagnoses formulated by Respiratory Physicians; postoperative changes in treatment; time taken for the initiation of treatment change and response to change in treatment.

Response to treatment was determined by analysing changes in symptoms, PFT performance, and imaging on Chest X Ray or CT scan. In most cases, changes in these clinical measures correlated, however, deterioration in any one of these clinical measures prevented the case from being considered as a “positive response to treatment”.

There were limits to the data on Trak-care system: PFT readings prior to VATS were only available for 55% of patients; data regarding size of biopsies were only available for 71% of patients; data concerning changes in treatment were available for 80% of patients; and data regarding response to treatment were available for 12 out of the 26 patients in which a change in treatment was initiated.

## Results

The characteristics of the 66 patient who underwent VATS are summarised in Table 1. 53% of the cohort were female and the average age of the cohort was 59 years. The commonest symptoms on first presentation were dyspnoea (73%) and a dry (36%) or productive (29%) cough. The average length of time between when a patient first presented with symptoms to when the VATS procedure took place was 16 months. The average FEV_1_ and FVC readings in patients prior to VATS were 2.11 L and 2.71 L respectively. Prior to VATS being performed, 25.8% of patients had already undergone BAL and 7.6% had undergone a TBLB, whilst steroid or immunosuppressive therapy had already been initiated or trialled in 16.7% of patients.

**Table 1:** Characteristics of patients

No. of patients: **66**		
Gender: Male: **47%**, female: **53%**		
Age: **58.9**		
Presenting symptoms:		
	Dyspneoa: **72.7%**	
	Dry cough: **36.4%**	
	Productive cough: **28.8%**	
	Pleuritic chest pain: **10.6%**	
	Haemoptysis: **6.1%**	
	Wheeze: **4.5%**	
	Systemic disturbance: **16.7%**	
	Asymptomatic: **4.5%**	
Time between first presentation and VATS lung biopsy: **16.1 months**		
Pre-operative pulmonary function testing:		
		FEV_1_: **2.11 L**
		FVC: **2.71 L**
Prior investigations:		
	Bronchoscopy: **33.3%**	
	Bronchoalveolar lavage: **25.8%**	
	Transbronchial lung biopsy: **7.6%**	
Pre-operative therapy (corticosteroid or other immunosuppressant): **16.7%**		

Characteristics of the VATS lung biopsy procedure are summarised in Table 2. More than one biopsy was taken in 42.2% of patients. The average biopsy size was 16.8 cm^2^. The most common sites for biopsy were: the left lower lobe (44%), the left upper lobe (41%) and the right lower lobe (28%).

**Table 2:** Number, site and size of VATS lung biopsies

Number > 1: **42.2%**	
Biopsy volume: **16.8 cm**^**2**^	
Biopsy site:	
	Left lower lobe: **43.8%**
	Left upper lobe: **40.6%**
	Right lower lobe: **28.1%**
	Right middle lobe: **17.2%**
	Right upper lobe: **3.1%**

Complications related to the VATS lung biopsy procedure are summarised in Table 3. There was one death (30 day mortality rate: 1.5%) in the patient cohort. The circumstances of this death were that the patient suffered a sudden deterioration in respiratory function 10 days after surgery, thought to be secondary to sepsis, and subsequently died on postoperative day 16; autopsy revealed diffuse alveolar damage and severe interstitial pulmonary fibrosis of unclassifiable type. There were no other major complications seen in the patient cohort. Minor complications were experienced in 28.8% of patients; these included small pneumothorax (10.6%), lower respiratory tract infection (6%) and surgical emphysema and prolonged neuropathic pain (both 4.5%). The average duration of time that a chest drain was left in situ was 1.15 days and the average hospital stay was 3.5 days.

**Table 3:** Mortality and morbidity of VATS lung biopsies

30-day mortality: **1.5%**	
Other major complications: **0%**	
Minor complications: **28.8%**	
	Small pneumothorax: **10.6%**
	LRTI: **6.1%**
	Surgical emphysema: **4.5%**
	Prolonged neuropathic pain: **4.5%**
	Delayed wound healing: **3%**
	Persistent air leak: **1.5%**
Conversion to open lung biopsy: **0%**	
Chest drain duration: **1.15 days**	
Hospital stay: **3.53 days**	

The most common differential diagnoses obtained from CT scanning were UIP (in 28.8% of patients), hypersensitivity pneumonitis (24.2%), NSIP (18.2%), infection, Cryptogenic Organising Pneumonia, sarcoidosis and Connective Tissue Disease (all 13.6%) (Table 4).

**Table 4:** Differential diagnoses given after CT imaging

**Ddx after CT scan**	**No. of patients**	**%**
UIP	19	28.8
Hypersensitivity pneumonitis	16	24.2
NSIP	12	18.2
Infection	9	13.6
COP	9	13.6
Sarcoidosis	9	13.6
CTD	9	13.6
Malignancy	8	12.1
No differential given	6	9.1
Drug	4	6.1
Other	22	33.3

A Definite Pathological Diagnosis (DPD) was achieved from VATS lung biopsy in 74.2% of cases (Table 5). The most common pathological differential diagnoses made were Hypersensitivity Pneumonitis (31.8%) and UIP (28.8%), followed by Connective Tissue Disease (CTD) (13.6%), NSIP (12.1%) and sarcoidosis (10.6%) (Table 6).

**Table 5:** Diagnostic accuracy of VATS lung biopsy and postoperative rates of therapy change

Definite Pathological Diagnosis (DPD): **74.2%**	
Change in treatment (overall): **47.2%**	
Change in treatment in “DPD” cohort: **46.2%**	
Change in treatment in “no DPD” cohort: **50%**	
Change in treatment in diagnoses of:	Hypersensitivity Pneumonitis: **80%**
	Sarcoidosis: **60%**
	NSIP: **40%**
	UIP: **9%**
Positive response to treatment: **58.3%**	
Time taken for change of treatment: **12.7 weeks**	

**Table 6:** Differential diagnoses given based on VATS biopsies

**Ddx of VATS biopsy**	**No. of patients**	**%**
Hypersensitivity pneumonitis	21	31.8
UIP	19	28.8
CTD	9	13.6
NSIP	8	12.1
Sarcoidosis	7	10.6
Aspiration	3	4.6
Pulmonary Langerhans Histiocytosis	3	4.6
Infection	3	4.6
Stoneworkers pneumoconiosis	2	3.0
End stage fibrosis	2	3.0
Other	21	31.8

Consensus diagnoses were formulated based on findings from the CT scans and lung biopsies, in addition to consideration of the overall clinical picture of each patient. The most common consensus diagnoses made were: Hypersensitivity Pneumonitis (22.2%), UIP (20.6%), sarcoidosis (9.5%) and NSIP and CTD (both 7.9%). In 9.5% of patients, the interstitial lung disease process occurring was deemed to be unclassifiable (Table 7).

**Table 7:** Consensus diagnoses given after VATS biopsies

**Consensus diagnoses after VATS biopsy**	**No. of patients**	**%**
Hypersensitivity pneumonitis	14	22.2
UIP	13	20.6
Unclassifiable	6	9.5
Sarcoidosis	6	9.5
NSIP	5	7.9
CTD	5	7.9
RBILD	3	4.8
COP	2	3.2
End stage fibrosis	2	3.2
Vasculitis	2	3.2
Other	14	22.2

A change in treatment was initiated in 47.2% of patients subsequent to VATS being performed. A change in treatment was initiated in: 80% of patients diagnosed with Hypersensitivity Pneumonitis; 60% of patients with sarcoidosis and 10% of patients with UIP (Table 5). A change in treatment was initiated in 46.2% of the patients who received a DPD and 50% of the patients who did not receive a DPD (Table 5). Therapy change was initiated on average 12.7 weeks after the VATS biopsy (Table 5). Among the patients who underwent a change in treatment, 58.3% demonstrated a positive response to treatment (Table 5).

**Figure 1:**
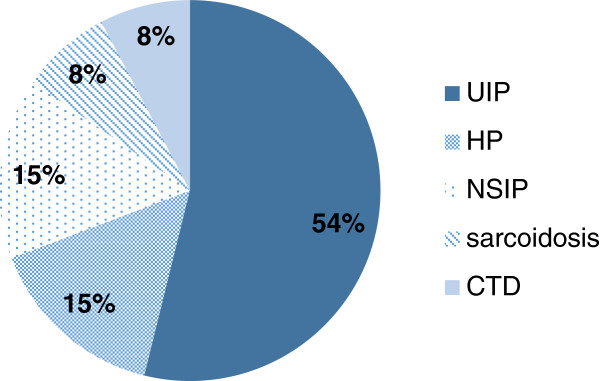
Proportional breakdown of postoperative consensus diagnoses given to CT differential diagnoses of “probable UIP”.

The differential diagnosis of “probable UIP” was reported in the CT scans of 13 patients. Only 54% of these patients received an eventual diagnosis of UIP, with 15% of them each having HP and NSIP (Table 8, Figure 1). 83% of patients considered to have probable HP at CT scan were eventually given a consensus diagnosis of HP. 33% of patients considered to have probable NSIP at CT scan were eventually given a consensus diagnosis of NSIP (Table 8).

**Table 8:** Correlation between CT scan differential diagnoses and post-VATS biopsy consensus diagnoses

**CT differential Diagnosis**	**Level of certainty**	**No. of cases**	**Consensus diagnosis (after VATS biopsy)**					**Correct diagnosis**
			**UIP**	**HP**	**NSIP**	**Sarcoidosis**	**Other**	
UIP	Probable	13	7	2	2	1	1	54%
UIP	Possible	6	3	1	1	0	1	50%
HP	Probable	6	0	5	0	0	1	83%
HP	Possible	9	5	2	0	2	0	22%
NSIP	Probable	3	0	0	1	0	2	33%
NSIP	Possible	9	4	2	2	0	1	22%
Sarcoidosis	Probable	3	0	0	0	2	1	67%
Sarcoidosis	Possible	6	0	0	0	3	3	50%

Proportional breakdown of postoperative consensus diagnoses given to probable/possible CT scan differential diagnoses of: UIP, Hypersensitivity Pneumonitis (HP), NSIP and sarcoidosis.

54% of patients given a consensus diagnosis of UIP, were considered as having “probable UIP” at CT scan (Table 9). 40% of patients given a consensus diagnosis of NSIP, and 14% of patients given a consensus diagnosis of HP were considered as having “probable UIP” at CT scan (Table 9).

**Table 9:** Correlation between CT scan differential diagnoses and post-VATS biopsy consensus diagnoses

**Consensus diagnosis (after VATS biopsy)**	**No. of cases**	**Probable diagnosis at CT scan**						
		**UIP**	**HP**	**NSIP**	**Sarcoidosis**	**Malignancy**	**Other**	**Ambiguous**
UIP	13	7 (54%)	0	0	0	0	2	4
HP	14	2 (14%)	5 (36%)	0	0	0	2	5
Sarcoidosis	6	1 (17%)	0	0	2 (33%)	0	0	3
NSIP	5	2 (40%)	0	1 (20%)	0	1 (20%)	1	0
Malignancy	1	0	0	0	0	0	1	0

Proportional breakdown of preoperative “probable diagnoses at CT scan” given to consensus diagnoses of UIP, Hypersensitivity Pneumonitis (HP), sarcoidosis, NSIP and malignancy.

## Discussion

Obtaining a specific diagnosis is a challenging process for many patients with ILD. CT scanning achieves a correct diagnosis in only 61 - 80% of cases of ILD [2], whilst the minimally invasive procedures, BAL and TBLB, have limited value in this diagnostic setting. Hence almost one third of patients with ILD will eventually require a Surgical Lung Biopsy (SLB) to obtain a definite diagnosis, with the procedure of choice in today’s surgical setting being Video-Assisted Thoracoscopic Surgery (VATS) [15-18].

Prior to VATS being performed, only 7.6% of patients in this study had undergone a Transbronchial Lung Biopsy (TBLB); this figure is comparable to that (11.9%) reported in a similar study by Qureshi *et al*. [19]. These figures reflect that for many cases of ILD, indeterminate in type after CT imaging, obtaining a TBLB is deemed to be unsuitable, in that the procedure is unlikely to yield a Definite Pathological Diagnosis (DPD); such cases are instead directly referred for a Surgical Lung Biopsy (SLB). TBLBs are only likely to yield specific diagnoses in cases whereby small specimens are expected to be diagnostic, and particularly in forms of ILD with bronchocentric involvement [20]. For instance, a much larger size of specimen than that obtained by a TBLB, is normally required to make the diagnosis of UIP [21,22].

Amongst our group of 66 patients, there was only one death as a result of the VATS procedure (30 day mortality rate: 1.5%). In fact, this was the only major complication encountered in the patient cohort. Other studies have reported similar low mortality rates for VATS (0% - 3.17%), when performed to obtain a diagnosis of ILD [19,23-26]. There have been studies which have reported higher mortality rates for a Surgical Lung Biopsy (SLB) being performed in this setting (4.8 - 24%), but all such studies have had relatively higher proportions of their patients undergoing Open Lung Biopsies (OLBs), a more invasive surgical procedure than VATS [27-30]. Minor post-operative complications were experienced in nineteen of our patients (28.8%), a figure which is higher than that reported in other studies. Kreider *et al.* reported an overall post-operative complication rate of 19.1% [25], whilst the minor post-operative complication rate was only 6.8% in the study by Blackhall *et al.*[27]. The vast majority of the nineteen patients who experienced minor post-operative complications in this study, did not have their stay in hospital prolonged as a result, and indeed the average hospital stay reported in our study was only 3.53 days. Therefore, on balance, the complete absence of any major post-operative complications (including mortality) in sixty five out of the sixty-six patients included in the study, gives us considerable reassurance about the relative safety of the VATS procedure in this setting.

Patients included in this study had been highly selected for: they all had forms of ILD which couldn’t be diagnosed on CT scan. In consideration of this fact, we would deem the diagnostic capabilities demonstrated by VATS in our study – in obtaining a DPD in around three quarters of cases – to be substantial. There is marked variation between what previous studies have reported the rate of DPDs being yielded by SLBs to be; figures vary between 34 - 100% [24,26,27,31-33]. A figure comparable to this study (69.9%) was recently reported by Blackhall *et al.*[27], whilst two other studies reported a DPD being obtained from 100% of the patients with suspected ILD, undergoing the VATS procedure [24,26]. It is worth noting, our data did not show there to be a reduced rate of therapy change among the 25.8% of patients with no DPD after VATS. This may indicate that even when VATS fails to provide a DPD, it can still be of great help to the clinician, in excluding various differential diagnoses proposed by prior CT imaging, and thus narrowing the diagnostic possibilities for a patient.

That VATS biopsy provoked a change in treatment in almost half of our patients gives strong suggestion that this procedure is of considerable benefit to patients with ILD. Other studies have reported similar numbers of patients having their therapy altered after SLB. In a recent study by Blackhall *et al.*, 45.6% of patients had their therapy altered [27], whilst Kramer *et al.* reported a change in therapy in 46% of its patients after SLB [34]. Fifty four per cent of patients with ILD had their therapy altered after SLB, in a report by Walker *et al.*[32]. An even higher percentage of patients received a change in therapy (84.2%) in their report of 196 patients by Lee *et al.*[30].

Our data also demonstrates there to be a clinical benefit for the majority of patients who have undergone a change in therapy after VATS: 58% of our patients demonstrated a positive response to treatment. A similar proportion of patients (63.3%) were shown to display a clinical improvement after SLB in the study by Lee *et al.* too [30].

However, it was not just by provoking an initiation of new treatment that long term management of these patients was aided by obtaining a VATS lung biopsy. We would also argue that VATS biopsy was of benefit to patients receiving a UIP diagnosis. UIP was the second commonest consensus diagnosis made amongst our patients. The progression of UIP has been shown to be unaffected by pharmacological intervention, and thus NICE currently does not recommend pharmacological intervention for most cases of UIP; the guidelines state: “there is no conclusive evidence to support the use of any drugs to increase survival of people with IPF” [35]. A change in treatment was initiated in only 9% of our patients diagnosed with UIP. Thus steroid therapy was withheld in the overwhelming majority of our patients who received a diagnosis of UIP. Crucially, only 54% of patients receiving a diagnosis of UIP after undergoing a VATS lung biopsy had originally been given a diagnosis of “probable UIP” at CT scan (Table 9). The VATS lung biopsy particularly served to benefit the remaining 46% of patients whose CT scans had not indicated “probable UIP”: if these patients had not undergone VATS lung biopsies, they may have ended up with alternative ILD diagnoses and as a result, been unnecessarily exposed to the toxic side effects of immunosuppressive therapy.

Whilst UIP was the most frequent differential diagnosis made at CT scan among our patients, it was only the second most common differential reported at VATS biopsy, and likewise the second most common disease assigned as a consensus diagnosis. Hypersensitivity Pneumonitis instead, became the foremost ILD type diagnosed in our patient cohort after VATS (Table 7). Hypersensitivity Pneumonitis, in contrast to UIP, is a form of ILD which does respond to steroid therapy [20]. Indeed steroid therapy was initiated in 80% of our HP patients post-VATS biopsy; this figure would have been even higher if it wasn’t for the fact that steroid therapy was contraindicated in one particular patient with osteoporosis.

Our data shows VATS biopsy to be useful in that it frequently manages to differentiate HP from UIP: 15% of CT cases which were considered as “probable UIP” on CT scan, were proven to be HP on histology (Table 8, Figure 1). Moreover, 14% of the cases of biopsy-proven HP had earlier been considered as “probable UIP” on CT scan (Table 9). There are certain radiological features which are particularly specific to HP (centrilobular nodules) or UIP (basal, subpleural, reticular opacities associated with traction bronchiectasis) and allow for their confident differentiation on CT scan. However, when these disease-specific radiological features are absent, and when radiological patterns common to both HP and UIP (honeycombing, ground glass attenuation) predominate on CT scan, it is difficult to distinguish between these two diseases with a high level of confidence. Lynch *et al.* found that the accuracy of distinguishing UIP from HP on CT scan falls from 90%, when the CT diagnosis is made with a high level of confidence, to 60%, when the features on CT scan don’t allow a high level of confidence [36]. Silva *et al.* reported that UIP can be confidently distinguished from HP (or NSIP) by CT imaging in only 53% of cases [37]. In a more recent study by Sverzellati *et al.*, thoracic radiologists retrospectively reviewed the CT scans of 55 patients with biopsy-proven UIP; a probable diagnosis of HP was made in 7% of these CT scans [38].

Non-Specific Interstitial Pneumonia (NSIP) is another variant of ILD which can often be difficult to distinguish from UIP on CT scan alone, and this was also shown in our data: 40% of the cases of biopsy-proven NSIP, had been considered as “probable UIP” on CT scan (Table 9). One study demonstrated an incorrect diagnosis of probable NSIP to be retrospectively made in 34% of patients with biopsy-proven UIP [3]. In a retrospective study of 55 patients with biopsy-proven UIP, by Sverzellati *et al.*, 33% of CT scans retrospectively analysed, were classed as probable NSIP [38]. Again, the clinical implications of being able to accurately distinguish NSIP from UIP are considerable: NSIP, as well as having a substantially better prognosis than UIP, is also a disease which is much more likely to be responsive to immunosuppressive therapy. Indeed steroid therapy was initiated in 40% of the patients who were given a consensus diagnosis of NSIP in our study.

## Conclusion

It is beneficial to obtain a VATS lung biopsy in patients with ILD indeterminate in type from prior investigation. With the deployment of this procedure, a specific diagnosis can be obtained in roughly three quarters of these patients, with a resultant change in treatment being prompted in almost half of these patients, including in the vast majority of Hypersensitivity Pneumonitis cases. It also ensures that patients with UIP aren’t given inappropriate immunosuppressive treatment. It is recommended that the VATS procedure is performed for all patients with ILD indeterminate in type from prior investigation.

## Competing interests

The authors declare that they have no competing interests.

## Authors’ contributions

DM participated in the design of the study and drafted the manuscript. VZ conceived of the study, and participated in its design. Both authors read and approved of the final manuscript.
